# Experiences and Perceptions of Nursing Students during the COVID-19 Crisis in Spain

**DOI:** 10.3390/ijerph181910459

**Published:** 2021-10-05

**Authors:** Julián Rodríguez-Almagro, Antonio Hernández-Martínez, Cristina Romero-Blanco, Alejandro Martínez-Arce, Maria del Carmen Prado-Laguna, Francisco Jose García-Sanchez

**Affiliations:** 1 Department of Nursing, Physiotherapy and Occupational Therapy, Ciudad Real Faculty of Nursing, University of Castilla-La Mancha, 13071 Ciudad Real, Spain; Julianj.rodriguez@uclm.es (J.R.-A.); Cristina.Romero@uclm.es (C.R.-B.); 2 Center of Advanced Simulation, Hospital of Ciudad Real, SESCAM, 13005 Ciudad Real, Spain; amarce@sescam.jccm.es; 3 Department of Nursing, Dean of the Nursing Faculty of Ciudad Real, University of Castilla La-Mancha, 13001 Ciudad Real, Spain; Carmina.Prado@uclm.es; 4 Ministry of Health, Community Board of Castilla la Mancha, 13001 Ciudad Real, Spain; fjgarcia@sescam.org

**Keywords:** COVID-19, nursing students, pandemic, perceptions, experiences, qualitative research

## Abstract

In the early stages of the 2019 coronavirus pandemic in Spain, the Spanish health system was overwhelmed, mainly due to a lack of personnel, and many hospital centers collapsed by following avalanche of patients with COVID-19; this meant that the National System of Health called for fourth-year nursing students to come to the hospital as health care personnel. Our aim was to describe the perceptions and experiences of a sample of nursing students during the early stages of the outbreak. We conducted a qualitative study with an empirical-phenomenological approach. Twenty nursing students in their final year of study in Spain were recruited using purposive and snowball sampling. They participated in in-depth interviews between 20 April and 10 May 2020. The interviews were transcribed and then analyzed using Haase’s adaptation of Colaizzi’s phenomenological method. Four main themes emerged from data analysis: “social responsibility and pride as a health worker”, “pressure caused by working with COVID-19 patients”, “feeling defenseless and let down”, and “personal growth as a health worker”. These main themes were further divided into 11 theme categories. Due to an intense work day for several days in a row, the students were tired and mentally exhausted. Even so, they managed to overcome any difficulties, demonstrating their professional dedication and resilience. Greater preparatory support should be provided to safeguard the well-being of these future healthcare providers. More intensive preparatory training is necessary for health sciences students to facilitate crisis preparedness and effective crisis management. It is necessary to implement support from healthcare systems, including sufficient personal protective equipment, as well as contracts that accurately reflect the work they do. It is necessary for nursing supervisors to have effective communication in the performance of their functions with nursing students; this dialogue helps to clearly explain which are the functions that students must perform when they are carrying out their internships. There is also a need for preparatory training in managing infectious diseases such as COVID-19.

## 1. Introduction

Coronavirus disease 2019 (COVID-19) is growing rapidly worldwide. On 11 March 2020, the World Health Organization (WHO) announced that COVID-19 had reached pandemic status [1]. COVID-19 has resulted in a global pandemic. The disease is associated with a series of clinical symptoms from asymptomatic infections to mild and severe manifestations, contributing to a significant morbidity and mortality [2]. Clinical reports from China have established that COVID-19 presents as an acute febrile respiratory illness as the dominant feature of a systemic disease involving multiple organ systems [3].

On 9 May 2020, 855,788 cases had been confirmed worldwide, with 275,862 deaths. In May of 2020, Spain was the worst affected country in Europe, both in terms of the number of confirmed cases (222,857) and the number of deaths (26,251) [4,5]. In Spain, the Carlos III Health Institute (ISCIII), through the National Network for Epidemiological Surveillance (RENAVE), publishes a weekly report on COVID-19 infections among health workers in Spain. Of the 217,543 total confirmed cases on 7 May 2020, 35,548 were health professionals and administrative staff (health professionals understood to be a heterogeneous group including hospital staff, medical center staff, etc.) [6]. One important aspect that the ISCIII report [6] analyses is that these infections among health workers soared beginning 6–7 March 2020, i.e., the early stages in which restrictive and physical distancing measures had not yet been adopted, as reported by various healthcare periodicals [7,8].

The rapid rate at which the disease spread overwhelmed the Spanish healthcare system, as occurred in other countries [4,9]. Spanish hospitals adapted and converted specialist units into COVID-19 units and created new COVID-19 units from scratch in order to cope with the very high number of patients with COVID-19 symptoms. As the epidemic progressed in Spain, more trained people were needed, capable of attending to the large numbers of people with COVID-19 symptoms [6]. A ministerial order published in the Spanish Official State Gazette on 15 March 2020 [10] established that undergraduate nurses could begin working provided that was this “to provide to support or under the supervision of a professional”. Similar measures were taken in other countries [11,12].

The reality, however, was quite different. The lack of resources meant that students without any work experience were put on the front line, with some public and private hospitals allocating students in emergency (A&E) and intensive care units (ICUs). While unions concede that this practice is not widespread, they warn that it was causing students unnecessary stress and devastating emotional trauma [13,14,15]. On top of this, students were being paid low salaries (around EUR 900 to 1200, depending on whether they were studying nursing) and had precarious contracts. We must state that the average salary of a registered nurse in Spanish public health ranges between EUR 1500 to 1900. Students did not know how long they would be on these contracts or whether or not they will be renewed [16]. These student nurses were recruited to work as clinical support workers, and not as qualified nurses. Currently, these types of contracts are not being carried out.

Students have been vital resources for the healthcare system. Their health and safety are crucial, not only because of the impact these have on patient health and safety, but because these students will play a key role in future pandemics.

The literature tells us that, in previous outbreaks of other diseases such as severe acute respiratory syndrome (SARS) and Middle East respiratory syndrome (MERS), health workers suffered from high amounts of stress related to the high risk of infection, stigma, as well as the lack of personnel and uncertainty. In these cases, integrated support was a high priority for the healthcare systems in question [12,17,18]. One study showed that caring for patients with COVID-19 carries with it a greater risk of mental health problems such as anxiety, depression, insomnia, and stress [19]. Due to their lack of previous experience, nursing students face the additional challenge of a new working environment and an unfamiliar disease.

According to our searches, no qualitative studies had been published on these experiences, which are necessary to understand. Our objective was to describe the perceptions and experiences of the nursing students that looked after patients with COVID-19 symptoms in the early stages of the outbreak. This study is framed within a research question centered around if there have there been problems in the experiences of nursing students during the COVID pandemic when dealing with these patients.

## 2. Material and Methods

### 2.1. Study Design

We conducted a qualitative study using interviews. We used a phenomenological-empirical approach to obtain detailed descriptions of the experiences of nursing students with no prior experience with COVID-19 patients. An inductive framework analysis approach was used to analyze data [20]. The phenomenological approach is based on the study of life experiences, regarding an event, from the subject’s perspective. This approach assumes the analysis of the most complex aspects of human life, of that which is beyond the quantifiable. According to Husserl, it is a paradigm that seeks to explain the nature of things, the essence, and the veracity of phenomena. The objective it pursues is the understanding of the experience lived in its complexity; this understanding, in turn, seeks awareness and meanings around of the phenomenon [21]. Throughout the study, we followed the COREQ Standards for Reporting Qualitative Research guidelines [22].

### 2.2. Participants and Study Location

All participants were in the final year of their degree when they were considered by the interviewers for this study. All participants received an explanation of the study objectives and the fact it was voluntary. Participants gave verbal consent prior to each videoconference interview. Confidentiality was ensured by using numbers instead of names (e.g., nurses N1, N2, etc.) and eliminating any identifying information from the transcriptions. All audio recordings and transcriptions were password-protected and kept on a secure computer. All audio recordings and transcriptions were stored with password protection on a protected computer.

Participants were recruited through purposive and snowball sampling [23]. We selected 20 nursing students who had been recruited by the health service to provide medical assistance and/or support to COVID-19 patients. The sample size was determined using the principle of saturation, which is when participants are added until no new categories emerge [24]; 10 participants were known to two of the interviewers and the remaining participants were recruited through snowball sampling.

The data became saturated at interview number eighteen. Two further interviews were conducted to confirm the redundancy of themes. The interviews lasted between 25 and 65 min. The researchers did not previously know the interviewees.

Our sample consisted of twenty recruited nursing students who contacted the research team through the sampling used and who were carrying out their work in Spanish hospitals with COVID-19 patients in April 2020. Five nursing students declined to take part in the study due to their anxiety of having to talk about what they were going through.

### 2.3. Data Analysis

The data collection method was semi-structured interviews conducted via video conference (Skype). In-depth semi-structured interviews were conducted in person and by video conference between 15 and 25 April 2020, at times chosen by the participants. The interviews were recorded with the permission of the participants. We collected data including age, gender, marital status, the department they had been allocated to work in as clinical support workers, and the number of days they had been working with COVID-19 patients.

All interviews began with an open-ended question: “Please tell me what caring for patients with COVID-19 is like for you”. They were then asked follow-up questions to encourage them to talk about their experiences to increase the depth of the responses (Table 1).

The data were analyzed as they were collected, that is, the interviews were transcribed and thematic categories were searched. The audio recordings were transcribed by two researchers and reviewed by a third researcher for additional accuracy. The interviews were conducted in Spanish by two members of the research team, and then the data analysis on original transcripts were carried out. All of the text was translated into English by a native translator then back-translated into Spanish by another translator to ensure that the meaning was the same. The final text was then validated by the researchers.

Haase’s adaptation of the Colaizzi method was used to analyze the transcriptions [25,26,27]. The analysis involved reading the transcriptions several times to understand the meanings conveyed, identifying significant sentences and reformulating them to validate the meanings agreed by the whole research team, identifying the themes and organizing them into clusters and categories, and giving a full description of the themes [27].

During the process, the criteria for methodological rigor of credibility, dependability, confirmability, and transferability were observed [28].

Credibility was achieved by in-depth interviews followed by peer debriefing [27,28,29]. Two co-authors analyzed the transcripts independently by bracketing data on preconceived ideas and strictly following the adapted Colaizzi method described above [27,28,29]. Findings were then compared and discussed by the team until consensus on themes, theme clusters, and categories was achieved [27,28,29]. Transferability was established by considering variations of participant characteristics and sufficient quotations collected through in-depth interviews [27,28,29]. The audit trail was maintained to ensure all analysis steps could be traced back to original interviews [27,28,29].

All authors participated in the validation of the results, questioning each step of the analysis to check any alternative interpretations. The analysis was discussed until agreement was reached.

### 2.4. Ethical Considerations

This study has received the approval of the Clinical Research and Ethics Committee from the Dean’s Committee of the Ciudad Real Faculty of Nursing at the University of Castilla la Mancha, with protocol number 04/2020.

## 3. Results

All participants were in contact with COVID-19 patients in COVID-19 units at the hospitals they were recruited to work at from mid-March. All of the participants are, at the time of writing, still at the units the interviews were conducted at (Table 2) and had spent between 10 and 40 days at these units.

Four main themes emerged from the analysis of the interviews (Table 3): “social responsibility and pride as a health worker”, “pressure caused by working with COVID-19 patients”, “feeling defenseless and let down”, and “personal growth as a health worker”. These clusters were further divided into 11 subtheme categories.

### 3.1. Social Responsibility and Pride as a Health Worker

All twenty participants voluntarily signed up to sign their contract as clinical support workers despite being aware that they would be working with COVID-19 patients. Furthermore, all twenty participants got involved at the start of the pandemic when the virus was spreading quickly and there was still a lot of uncertainty.

“I did it voluntarily because I wanted to help”

(N4).

“I am proud and happy that I took the decision to volunteer to work as a nursing auxiliary”

(N14).

“We took part voluntarily”

(N1).

“I signed up voluntarily because I wanted to help”

(N15).

#### 3.1.1. Gaining Work Experience and Enhancing Learning

The participants wanted to gain work experience and increase their learning, which they achieved with the support of qualified personnel.

“The experience has been quite enriching on both a personal and professional level. I have personally been quite lucky as far as the department and the professionals I have been working with.”

(N5).

“It has been a very gratifying experience as far as the help I have been able to offer and all of the experience I have gained”

(N17).

“It has been great because I have gained work experience that I could only have gained in these critical conditions.”

(N20).

“I have been thrown in at the deep end, learning as I go, and now, after 41 days, I have experience that it would have taken me months to obtain otherwise. I am still learning so much every day, I think I have learned more than I ever would have on any master’s course.”

(N10).

“It has been a one-of-a-kind experience that will have a positive impact on my learning and on my future as a nurse”

(N9).

“It has been a very positive experience, another learning experience under my belt”

(N3).

#### 3.1.2. Fighting COVID-19

Some participants saw this voluntary experience as gratifying because they got the chance to be on the front line in the fight against COVID-19.

“…feel useful and feel like I am doing my bit to fight COVID-19.”

(N16).

“I have worked as a COVID-19 operator, fighting this disease over the phone by detecting lots of cases and quarantining people to prevent more infections”

(N7).

“I continue fighting in the COVID-19 ward that was opened”

(N11).

#### 3.1.3. Pride as a Nurse

The participants saw attending to patients with COVID-19 as necessary despite the danger posed by the virus, with some of them considering this task to be an inevitable part of their role as future nurses and seeing it as their responsibility.

“I am proud I took the decision to volunteer to work as a medical auxiliary”

(N15).

“In these kinds of situations, you realize what your true calling is and feel proud at having chosen this profession.”

(N2).

“At the start it was terrible, then as the days went by I saw the situation improve and I got to do my part without getting infected, I feel proud and satisfied.”

(N6).

### 3.2. Pressure Caused by Working with COVID-19 Patients

Recurrent themes in participants’ responses include feelings of stress, fear, and sadness due to the emotional exhaustion brought about by the pressure of having to provide proper care. It takes a lot of effort to minimize complications in manipulating the airway, medication, patient positions, correctly putting on and taking off PPE, and providing quality care while avoiding infection. This caused them to feel afraid about getting infected and subsequently infecting their loved ones.

“We have experienced psychological pressure due to working with COVID-19 patients and everything that that carries with it”

(N12).

“I think that the hardest thing about this experience is definitely the psychological fatigue and the stress we are exposed to. Because we are in an unprecedented situation”

(N18).

“It has been the worst experience because I have been in situations that have made me feel bad, guilty, anxious, nervous, incompetent and out of place. Despite everything.”

(N19).

#### 3.2.1. Emotional Lability and Psychological Exhaustion

Participants told us that their experiences were very difficult on an emotional level. As they were still students, they felt uncertainty and psychological fatigue, and were worried about feeling out of place or seeming incompetent.

“Comments from certain colleagues referring to us as being a ‘hindrance’ or ‘useless’ also had a negative impact. I only heard it once and from someone that had never seen me working, but it stuck with me for a while.”

(N13).

“With the emotional lability and stress I faced every day, it was the last thing I needed to hear.”

(N8).

“I am on a COVID-19-only ward and that has affected me emotionally.”

(N3).

“It has been a very hard experience emotionally more than professionally, due to the pressure to do everything well and have everything under control.”

(N5).

“It has been a very stressful situation with a lot of anxiety caused by not feeling sure about knowing how to handle patients.”

(N18).

“I felt uncertain about certain techniques and situations.”

(N11).

“I also felt very sensitive, despite feeling well overall, there were times of day when anything I saw on the news made me cry, sometimes it was because of people’s solidarity, other times because I think I was more conscious of the situation, all of the awful news.”

(N8).

“Lots of stress and days when you didn’t even know who you were. Constant crying and sadness because of numerous deaths and knowing there was nothing you could do in those situations.”

(N16).

“I have experienced very sad situations like finding a patient had died in their room without being able to say goodbye or be with their family.”

(N2).

“It has been very stressful and demanding and very hard, we have had lots of patients die, but we have done everything we can.”

(N7).

#### 3.2.2. Fear of Being Infected Due to Poor Conditions

Some participants told us they are afraid of being infected due to poor conditions, a lack of equipment, and having to wear the same PPE multiple times.

“It was a very stressful situation for me because of the conditions we faced.”

(N6).

“I was afraid of becoming infected due to the lack of equipment”

(N10).

“At the start, I was very fearful because when I got to the ward, my duties were different to the ones I had been told I would be doing as stated in my contract”

(N19).

“I think it has been a very good experience, but under very difficult conditions.”

(N10).

#### 3.2.3. Fear of Infecting Family Members

Our participants felt afraid because of the unfamiliarity of the situation and the infectiousness of the virus. They wanted to work, but did not want to infect their families. In fact, many of our participants continue to live away from their usual home after leaving to avoid infecting their loved ones.

“It’s horrible not being able to hug my family even though we are living in the same house. My biggest fear is infecting them.”

(N17).

“Many of us, me included, have had to leave home to avoid putting our families in danger.”

(N9).

### 3.3. “Feeling Defenceless and Let Down”

Since the start of the pandemic, many of our participants have felt let down by the health system, and for being misled about shifts, pay, contracts, and responsibilities.

They have also felt alone and defenseless, often finding themselves working alone without anyone to supervise them.

They also report not being able to work properly due to a lack of equipment.

“I am really disappointed about the conditions we are working in. I feel let down and that right from the start they have avoided telling us the truth about what we would be doing.”

(N1).

“There has always been a lack of personal protective equipment.”

(N4).

“I am very happy that I am able to help despite the disastrous conditions.”

(N10).

“We have felt rejected to some extent because a lot of the staff felt that, as students, we were going to ‘waste material’ rather than be of any help.”

(N12).

#### 3.3.1. Let Down by the System (Contracts, Shifts, and Pay)

Nursing students reported feeling let down by the health system. They worked night shifts and holidays without being paid, and were promised working conditions and contracts that were still not materialized or that were totally different to the ones promised.

“The pay has been low considering the work we have put in, as we have been told we will not be paid for night shifts or holidays that we have worked.”

(N13).

“We have been continually let down from the start. We were not supposed to be on the front line, but a resus area with critical patients obviously is the front line. We weren’t supposed to be responsible for patients, but then after one week this changed and we were assigned patients, without any kind of supervision. We were going to be paid for the nights and holidays we worked, something that hasn’t happened either. I still don’t know what my health and safety conditions are if something happens with one of my patients. When you ask around at the hospital, nobody knows anything, or nobody wants to say anything. HR even told me that if I had any questions about my contract, and I quote, ‘to look it up online’. I understand that this is a critical and unprecedented situation, but I think they have taken advantage of us 4th year nursing students.”

(N6).

“We have been made to work shifts that we aren’t going to be paid for, and they are now telling us we will get days off in lieu when we were told they would be paid. We have had to demand to see our contract after asking numerous times, and we have seen that the conditions on the contract do not correspond to those we have been working under.”

(N14).

“All of this for a disgraceful salary considering the work we have done and the viral load we have been exposed to.”

(N20).

“The whole month I have been working, they have been telling us we would be paid for nights and holidays, something which they clearly aren’t going to do, apparently we will get time off in lieu instead.”

(N12).

“We were supposed to get paid for nights and holidays like everybody else, but now that the month is over and we have already worked a load of nights and holidays they’re telling us we won’t be paid and nobody knows why or who took the decision...”

(N8).

“After almost one month, they still haven’t specified any of our employment conditions in terms of health and safety, remuneration, job bank scores or responsibilities. We began working as auxiliaries and quickly began working as nurses (without the same working conditions nurses have).”

(N10).

“We were told we would be paid for nights and holidays, something that has NOT happened. I have worked three nights and three holidays this month. We aren’t going to be compensated for them at all (not even in days in lieu, which they should be giving me).”

(N13).

#### 3.3.2. Feeling Alone and Defenseless without the Supervision of Qualified Staff

Participants allude to feeling alone when working without the supervision of qualified personnel. Often, having been hired as medical auxiliaries, the participants ended up working as nurses without the proper supervision that they were told they would receive at the start of the pandemic.

“There were days I was solely responsible for patients and days I was being “supervised”, but with all of the chaos they were hardly going to be by my side all of the time.”

(N15).

“They told me it was for resus, a department that is now an ICU for COVID-19 patients, and I have been working like any other nurse, being allocated patients like everybody else, without anyone ever checking medications or incidents at any time, although they were always at hand to answer any questions I had. Once I went inside with PPE, which I did along with the rest of the nurses, the only supervision I had was from a nurse that was on the outside, who didn’t even know she was supposed to be supervising my work.”

(N5).

“It has been bitter-sweet, I really liked interacting with patients, caring for them and so on. But I didn’t like the sarcastic, dismissive attitude that some staff (a small minority) have toward students.”

(N17).

“The experience has been totally different. Initially, the authorities told us we weren’t going to be on the front line, something that has turned out to be false as ALL of us have been on the front line, some of us more exposed than others. We were never supervised.”

(N3).

“I went from being a student on placement to being solely responsible for a critical patient. Faced with that situation, there were lots of times the first few days I would get home in tears just wanting to go straight to bed. I suppose it would have been the same if I had graduated normally and started working as a qualified nurse for the first time.”

(N9).

Not all of the students talk about feeling defenceless, some felt supported by all of the staff and said that they received continued help and assistance.

“Our role was to provide support in the ICU. We were there as an extra pair of hands, as were some nurses, but there were always at least 6 nurses and then us. I was quite free to get stuck in with my duties a nurse, but I never felt alone or unsupported.”

(N2).

“Luckily, my time at the ICU has been rewarding. I am thankful for the great team I work with; they have taught me so much in a short space of time and have always been willing to help me. It’s true that I haven’t had close supervision, but the team has always been organized so that there is at least one experienced member of staff near a trainee.”

(N8).

“At the start I was really, really nervous because I didn’t know if the conditions of the contract would really be met (especially concerning supervision, indemnity insurance and so on). I gradually began to relax, especially because they sent me to the same ward I had already done my placement at.”

(N14).

### 3.4. “Personal Growth as a Health Worker”

Once the early stages of the pandemic had passed and as time went on, the students reported feeling more confident with the situation and feeling as though they were contributing to society, while also developing personally and professionally.

#### 3.4.1. Personal Satisfaction from Working during a Global Pandemic

Participants felt that they were growing both personally and professionally, which has led them to live it as an incredible experience in all aspects.

“I thought I would go home in tears every day, to be honest, but I have come out of it feeling motivated, because I know that my colleagues are there for me in an emergency or if I have any questions. It has been a very tough experience, but it has been very gratifying on both a personal and professional level. I would do it again.”

(N18).

“I am really happy that I decided to help during this difficult time.”

(N19).

“I am really happy that I have been able to help, I feel really useful and I am very satisfied with how things are going.”

(N4).

“Now that everything is a lot calmer, what has stayed with me has been the positive things the nurses I work with day in day out have said to me, and the fact they appreciate and value my work and effort during this difficult situation.”

(N1).

“A lot of personal satisfaction, as it has been fantastic to be able to help when everything has been so overwhelmed, especially working with such great professionals that work as a phenomenal team despite the disastrous conditions.”

(N6).

“It has been worth it, getting the chance to do our part to help. An incredible experience despite the conditions we began working in.”

(N11).

#### 3.4.2. Gaining Independence as a Health Worker

Participants believed that working during the pandemic helped them to improve all their skills as future health professionals.

“This experience has allowed me to learn a lot and help me prepare professionally. In a month’s time I will be working as a nurse.”

(N7).

“I am very happy because I have learned a lot, I haven’t felt afraid of getting infected and I haven’t felt like a burden. The team have taken me on as one of their own, for both the good times and the bad.”

(N5).

“I have got along well with all of the staff and the way of working. I have learned quite a lot and they have trusted in my ability to be a good professional.”

(N10).

“It has been a unique experience, since from my point of view, I feel more prepared and better trained than I did before. Although they misled us a bit, it is an experience I will never forget because of all of the new situations we have experienced, both good and not so good.”

(N2).

“I am learning a lot and feel like just another nurse on the team.”

(N14).

“The situation in the hospital was tough and I didn’t feel prepared, but with the help of my colleagues in the department I have gained experience and feel glad to help, despite everything I have mentioned.”

(N18).

“It has been the best experience of my life because I have gained work experience that I could only have gained in these critical conditions.”

(N15).

#### 3.4.3. Being Tested and Surpassing Oneself

The lived situation made some of the participants feel it as personal growth, both professionally and personally, which has led them to be happier in their lives.

“I am happy. I was able to help without being exposed to any risks, the situation has made me grow as a person and I have overcome my fears around working.”

(N12).

“I have learned a lot over the past month, and I am very happy to have had this opportunity.”

(N3).

“It has been an amazing experience. I think it has been the best experience of my life on a professional level and one of the best on a personal level, because it is really changing my way of seeing life, I get angry less, I only focus on the truly important things, I don’t worry about things that aren’t important but used to seem like the be all and end all...”

(N16).

“I am really grateful that I have been able to help, I feel fulfilled and very happy.”

(N19).

“I am grateful to have had the chance to do my part during this situation, which I hope is over very soon.”

(N7).

“I am very happy to be getting this experience. I am learning a lot at all levels, and although the first days were tough, I would make the same decisions.”

(N10).

## 4. Discussion

This study explores the first-person experiences of nursing students who were in the first line of care in hospitals in Spain, a country which has been greatly affected by the pandemic. This study contributes the shared reflections of 20 nursing students who, despite living in different cities, and having cultural and institutional differences, were facing direct care from people infected with COVID-19.

COVID-19 is a new, highly contagious virus which has spread very rapidly worldwide, particularly in Spain [4,5]. Despite this, the participants in our study did not hesitate to go and support their country, seeing it as a personal and professional challenge, even when there was a risk of becoming infected [30,31]. Other studies show us that health workers experience more pressure when caring for infectious patients; however, our participants did not see this as a reason to turn down medical auxiliary contracts as they feel responsible due to their status as nursing students [30,32], considering it an ethical and moral duty [33].

Students working with COVID-19 patients experience psychological stress, anxiety, and fear while caring for their patients, which is similar to the findings of similar studies involving infectious diseases [29,34,35,36].

Among our participants, there is a conflict between the duty to help and the fear of infection, due to the strict guidelines that professionals must follow in order to avoid becoming infected or infecting others. This discordance was observed in an existing study [37] in which it was concluded that, in response to a disaster such as a pandemic, when health workers’ safety, lives, and health may be at risk, it may be necessary to question whether healthcare organizations would be able to adequately fulfil their obligations. In our study, many nursing students share the concerns experienced by the general public regarding the unknown and what lies ahead for themselves, their patients, their colleagues, and their own families and friends. The global nature of this crisis means that, while all countries are engaged in the battle against COVID-19, some have been in the fight longer and, therefore, there is an opportunity to learn from other countries [38]. While dealing with the social changes and emotional stressors that all people face, nurses struggle with the increased risk of exposure, extreme workloads, moral dilemmas, and a rapidly changing practice environment that differs greatly from the day-to-day reality they are familiar with [11,39].

Due to the lack of equipment and the precarious conditions the students were working under, some of them felt defenseless and misled by the health system, which at the start of the pandemic offered them certain employment conditions that did not materialize or that turned out to be much worse than those promised [11].

Students sometimes noted a lack of supervision or less supervision than normal. This was made worse by the problems posed by COVID-19 patients, which qualified professionals must resolve quickly without necessarily having the time to explain to students what they are doing. Even when students did receive adequate training [40], they were sometimes afraid of harming patients [12].

The arrival of a pandemic and the need for personnel offered the students the possibility to begin working, something they would not normally have had the chance to do until the end of their studies. In this regard, and as mentioned in the study by Swift et al. [12], students felt that they were a part of history, learning and acquiring new skills and rising to a challenge which they saw as personally fulfilling and professionally worthwhile. Even after adapting to COVID-19 work, participants felt identity crisis as nursing students and anxiety living in the community. The gap between the perceptions and the capability to work in a COVID-19 ward might impair nurses’ competence, leading to identity crisis around continuing work or education [41,42,43].

### Strengths and Limitations

The use of snowball sampling is a notable limitation since the results are not generalizable, but they still help us to understand the phenomenon. This study has analyzed the perceptions of students who have worked on the frontline in hospitals units, emergency services, and intensive care units caring for critical and semi-critical patients infected with COVID-19. Future studies should analyze this phenomenon in other locations, such as private hospitals, health centers, and nursing homes.

We should also recognize that this is a small study, and we cannot generalize the findings. Although in qualitative research, a small sample size allows full exploration of participants’ experiences and in-depth analysis [44], and they provide a snapshot that can guide future initiatives for change.

## 5. Conclusions

The COVID-19 pandemic has led health services to increase nurse numbers and offer work to nursing students. The rapid spread of the virus, along with the lack of equipment and substandard employment conditions, have led to students becoming physically and mentally exhausted, and we must not forget that they are the future guarantors of public health.

We must ensure that students make the decision whether they want to collaborate as a health aid in future pandemics, and no matter what this decision is, health systems have a duty to ensure their physical and psychological safety.

It is necessary to implement support from healthcare systems, including sufficient personal protective equipment, contracts that accurately reflect the work they do, and not being misled in terms of pay. There needs to be effective communication from supervisors while carrying out their duties, and there is also a need for preparatory training in managing infectious diseases such as COVID-19.

## Figures and Tables

**Table 1:** Interview guide.

1. Please tell me what caring for patients with COVID-19 is like for you.
2. How did you feel on your first day with COVID-19 patients?
3. How do you feel now?
4. What challenges have you faced working with COVID-19 patients?
5. Do you think you have been able to resolve them?
6. Have you received help from your colleagues?
7. Please tell me more about that

**Table 2:** Participants Characteristics.

	Age in Years	Gender	Marital Status	Unit They Work at	Days Working with COVID-19 Patients
Nursing Student 1	21	Female	Single	A&E	14
Nursing Student 2	24	Female	Single	ICU	22
Nursing Student 3	23	Female	Single	COVID-19 unit	24
Nursing Student 4	22	Male	Single	COVID-19 unit	38
Nursing Student 5	30	Female	Married	Resus	36
Nursing Student 6	21	Male	Single	Surgery	11
Nursing Student 7	24	Female	Single	A&E	27
Nursing Student 8	23	Male	Single	ICU	5
Nursing Student 9	35	Female	Married	COVID-19 unit	31
Nursing Student 10	26	Female	Single	A&E	30
Nursing Student 11	22	Female	Single	COVID-19 unit	20
Nursing Student 12	23	Female	Single	COVID-19 unit	14
Nursing Student 13	21	Male	Single	COVID-19 unit	38
Nursing Student 14	22	Female	Single	COVID-19 unit	40
Nursing Student 15	24	Male	Single	A&E	17
Nursing Student 16	36	Male	Married	A&E	18
Nursing Student 17	34	Male	Married	ICU	7
Nursing Student 18	25	Female	Single	ICU	32
Nursing Student 19	26	Female	Single	COVID-19 unit	21
Nursing Student 20	26	Male	Single	COVID-19 unit	16

COVID-19 = Coronavirus Disease 2019.

**Table 3:** Main theme and subtheme categories.

**1. Social responsibility and pride as a health worker**
1.1. Gaining work experience and enhancing learning
1.2. Fighting COVID-19
1.3. Pride as a nurse
**2. Pressure caused by working with COVID-19 patients**
2.1. Emotional lability and psychological exhaustion
2.2. Fear of being infected due to poor conditions
2.3. Fear of infecting family
**3. Feelings defenseless and let down**
3.1. Let down by the system (contracts, shifts, and pay)
3.2. Feeling alone and defenseless without the supervision of qualified staff
**4. Personal growth as a health worker**
4.1. Personal satisfaction at having worked during a global pandemic
4.2. Gaining independence as a health worker
4.3. Being put to the test and surpassing oneself
